# A Lack of Effectiveness in the ATM-Orchestrated DNA Damage Response Contributes to the DNA Repair Defect of HPV-Positive Head and Neck Cancer Cells

**DOI:** 10.3389/fonc.2022.765968

**Published:** 2022-05-31

**Authors:** Sabrina Köcher, Henrike Barbara Zech, Leonie Krug, Fruzsina Gatzemeier, Sabrina Christiansen, Felix Meyer, Ruth Rietow, Nina Struve, Wael Yassin Mansour, Malte Kriegs, Cordula Petersen, Christian Betz, Kai Rothkamm, Thorsten Rieckmann

**Affiliations:** ^1^Department of Otorhinolaryngology, University Medical Center Hamburg-Eppendorf, Hamburg, Germany; ^2^Department of Radiotherapy and Radiation Oncology, University Medical Center Hamburg-Eppendorf, Hamburg, Germany; ^3^Mildred-Scheel Cancer Career Center HaTriCS, University Medical Center Hamburg-Eppendorf, Hamburg, Germany; ^4^ Research Department, Cell and Gene Therapy, Department of Stem Cell Transplantation, University Medical Center Hamburg-Eppendorf, Hamburg, Germany

**Keywords:** head and neck cancer, human papillomavirus (HPV), ataxia telangiectasia mutated (ATM), DNA damage response (DDR), radiation sensitivity, DNA double-strand break repair

## Abstract

Patients with human papillomavirus-positive squamous cell carcinoma of the head and neck (HPV+ HNSCC) have a favorable prognosis compared to those with HPV-negative (HPV−) ones. We have shown previously that HPV+ HNSCC cell lines are characterized by enhanced radiation sensitivity and impaired DNA double-strand break (DSB) repair. Since then, various publications have suggested a defect in homologous recombination (HR) and dysregulated expression of DSB repair proteins as underlying mechanisms, but conclusions were often based on very few cell lines. When comparing the expression levels of suggested proteins and other key repair factors in 6 HPV+ vs. 5 HPV− HNSCC strains, we could not confirm most of the published differences. Furthermore, HPV+ HNSCC strains did not demonstrate enhanced sensitivity towards PARP inhibition, questioning a general HR defect. Interestingly, our expression screen revealed minimal levels of the central DNA damage response kinase ATM in the two most radiosensitive HPV+ strains. We therefore tested whether insufficient ATM activity may contribute to the enhanced cellular radiosensitivity. Irrespective of their ATM expression level, radiosensitive HPV+ HNSCC cells displayed DSB repair kinetics similar to ATM-deficient cells. Upon ATM inhibition, HPV+ cell lines showed only a marginal increase in residual radiation-induced γH2AX foci and induction of G2 cell cycle arrest as compared to HPV− ones. In line with these observations, ATM inhibition sensitized HPV+ HNSCC strains less towards radiation than HPV− strains, resulting in similar levels of sensitivity. Unexpectedly, assessment of the phosphorylation kinetics of the ATM targets KAP-1 and Chk2 as well as ATM autophosphorylation after radiation did not indicate directly compromised ATM activity in HPV-positive cells. Furthermore, ATM inhibition delayed radiation induced DNA end resection in both HPV+ and HPV− cells to a similar extent, further suggesting comparable functionality. In conclusion, DNA repair kinetics and a reduced effectiveness of ATM inhibition clearly point to an impaired ATM-orchestrated DNA damage response in HPV+ HNSCC cells, but since ATM itself is apparently functional, the molecular mechanisms need to be further explored.

## Introduction

Squamous cell carcinoma of the head and neck (HNSCC) consists of two biologically distinct entities, which can be separated by the presence or absence of high-risk types of human papillomaviruses (HPV). Patients with HPV-positive (HPV+) tumors possess a markedly better prognosis as compared to patients with HPV-negative (HPV−) tumors (1–3). This favorable outcome is especially well established for oropharyngeal squamous cell carcinomas (OPSCC), which constitute the vast majority of HPV+ HNSCC. One of the underlying reasons for the favorable outcome of patients with HPV+ OPSCC is an enhanced sensitivity towards ionizing radiation, which is evident from patient cohorts treated with sole radiotherapy (RT) (4, 5). In line with the enhanced tumor radiosensitivity, HPV+ HNSCC cell lines possess enhanced radiation sensitivity *in vitro*, caused by a defect in DNA double-strand break (DSB) repair (6), which is concordantly observed when comparing patient-derived tumor slice cultures of HPV+ and HPV− OPSCC (7).

In recent years, various mechanisms have been implicated in the impaired DNA DSB repair capacity, but the results are partly conflicting. A defect in homologous recombination (HR) has been repeatedly suggested since different groups observed a failure of HPV+ HNSCC cells to form Rad51 foci. This was mechanistically explained by a decreased expression of the Rad51 loading factors BRCA2 or Cyclin D1, with the latter being a consequence of the high p16 levels in these cells (8–10). p16 was further reported to cause a decrease in TRIP12 expression and subsequently an increase in the ubiquitin ligase RNF168, which was also described to result in defective DSB repair, presumably by interfering with HR (11, 12). Intriguingly, the E7 oncoprotein from high-risk HPV-types was recently shown to bind RNF168 and impede its function at DSBs, leading to enhanced repair by HR (13). Furthermore, a lack of responsiveness to the TGF-β pathway was described, resulting in miR-182 mediated reduction in the protein levels of BRCA1 and the ATM-activating factor FOXO3. As a consequence, HPV+ cells showed a decrease in HR and an increase in the error-prone alternative endjoining (alt-EJ) pathway (14). Again intriguingly, for HPV-induced cervical cancer just the opposite mechanism, a TGF-β/Smad4-mediated increase of miR-182 has been reported and in breast cancer TGF-β was described to mediate BRCAness and PARP sensitivity by induction of miR-181, which was also reported to reduce the expression of BRCA1 and ATM (15, 16). Furthermore, the long isoform of miR182 (miR182-5p) was recently suggested as associated with inferior outcome in p16+ OPSCC (17).

Regarding the proposed DSB repair pathway choice, Leeman et al. confirmed a stronger use of alt-EJ based on a comparison of specific signatures in the genome sequences of HPV+ and HPV− HNSCC annotated in the TCGA database but did not find equivalent hints for an HR defect. Instead, they suggest an impairment of classical NHEJ (c-NHEJ) based on reporter gene assays with ectopic E7 expression (18). A reduced efficacy of cNHEJ has also been suggested by previous studies, in part based on the reduced expression of crucial NHEJ components, such as DNA-PKcs, Ku80, and 53BP1 (9, 19), and further reports describe a reduced expression of the phosphatidylinositol 3-kinase-related kinase SMG1 (20, 21). While a severe deficiency in base excision and single-strand break repair can also cause enhanced radiation sensitivity, components of these pathways, which in part are also critically involved in alt-EJ, were reported to be rather upregulated in HPV+ HNSCC and, in line with this, the pathways were reported to be functional. (18, 19, 22). Finally, in contrast to some of the findings above, Wallace et al. described an HR defect based on enhanced, instead of reduced, Rad51 foci numbers in keratinocytes expressing the major HPV16 oncoproteins E6 and E7 (23) and Bhide et al. also reported an HR defect based on enhanced Rad51 foci numbers, but in this case not after radiation but in patient samples taken one day after induction chemotherapy treatment (24).

In summary, an enhanced radiation sensitivity and impaired repair of radiation-induced DSBs in HPV+ HNSCC cells is well accepted, but the mechanisms suggested to be responsible for the repair defect are so far highly controversial.

## Material and Methods

### Cells and Cell Culture

All cell lines were grown in RPMI (Sigma-Aldrich) supplemented with 10% fetal bovine serum (FBS) (Biochrom AG) at 37°C, 10% CO2 and 100% humidification. HPV+ HNSCC cells UD-SCC-2, UM-SCC-47, UPCI-SCC-154 and HPV− cells HSC4 and SAT were described previously (6). Cell line identity was verified by a short tandem repeat multiplex assay (Applied Biosystems). ATM inhibition was generally performed using 10 µM KU55933 (Merck). PARP inhibition was performed using olaparib (Selleckchem) at the doses indicated.

### X-Irradiation

Cells were irradiated at room temperature with 200 kV X-rays (Gulmay RS225, Gulmay Medical Ltd.; 200 kV, 15 mA, 0.8 mm Be + 0.5 mm Cu filtering; dose rate of 1.2 Gy/min).

### Colony Formation Assay

Long-term cell survival and reproductive capacity was determined using a colony formation assay. PARP inhibition: Exponentially growing cells were seeded in defined numbers and after 4 h treated with olaparib as indicated. Two week after treatment the medium was exchanged and cells were incubated without inhibitor until the formation of colonies (1–3 further weeks, depending on cell line). Radiation: Exponentially growing cells were seeded in defined numbers into T25 cell culture flasks and after 4 h treated with an ATM inhibitor and irradiated after 30 min of incubation. Twenty-four hours post irradiation the medium was exchanged and cells were incubated without addition of inhibitor. Incubation time until colony formation varied between cell lines from 2 to 4 weeks. Irradiated samples of HPV+ cell lines were allowed to grow for an extended period, as the colony formation was apparently delayed.

In both assays, the number of colonies containing more than 50 cells was assessed. In the case of UM-SCC-47, feeder cells (UM-SCC-47; 20 Gy) were added to a total of 5,000 cells per flask to support plating efficiency. For UPCI-SCC-154 and SAT, the medium was changed to a 1/1 mixture of RPMI/10% FBS and Amniomax C-100 medium/7.5% Amniomax Supplement (both Gibco)/7.5% FBS two weeks after the end of treatment to facilitate colony formation. A single experiment always contained the full set of substances and radiation doses.

### Protein Extraction and Western Blotting

Comparison of repair protein expression: Exponentially growing cells were harvested in RIPA-buffer (Cell Signaling) supplemented with Halt™ Protease and Halt™ Phospatase Inhibitor Cocktail (Thermo Scientific). Protein concentrations were assessed using the BCA assay (Sigma), and polyacrylamide gels were loaded with 25 µg protein per sample.

Assessment of radiation-induced phosphorylation: 500,000 exponentially growing cells were seeded in T25 cm^2^ cell culture flasks and irradiated the following day with 6 Gy. In the case of ATM inhibition, the inhibitor or solvent (DMSO) was added 30 min before irradiation. At the indicated time points after radiation, the medium was exchanged with ice cold PBS and the cells were briefly stored on ice until direct harvest in SDS sample buffer.

Proteins generated from both procedures were detected by Western blot according to standard protocols using fluorescence imaging (LI-COR Odyssey CLx). A list of all primary antibodies used in the Western blot experiments is provided in **Supplementary Table 1**.

### Cell Cycle Assessment

Cells were harvested by trypsinization, fixed with 70% ethanol, briefly washed with PBS/0.2% Triton X-100 and subsequently incubated with PBS/0.2% Triton X-100/DAPI (4′,6-Diamidin-2-phenylindol, 1 µg/ml) for 30 min at room temperature in the dark. Cells were washed once with PBS/0.2% Triton X-100 and flow cytometric analysis was performed using a MACSQuant10 with MACSQuantify Software (Miltenyi Biotec). The portion of cells in the respective cell cycle phases was calculated using ModFit LT™ software (Verity Software House).

### Immunofluorescence

DSB repair kinetics: Exponentially growing cells on glass cover slips were fixed with PBS/4% formaldehyde for 10 min. Permeabilization and blocking were performed for 1 h or overnight with PBS/1% BSA/0.2% Triton X-100. The cells were subsequently incubated for 1 h at room temperature with the primary antibodies (mouse anti-gH2AX (clone JBW301, Merck); rabbit anti-53BP1 (#NB100-304, Novus Biologicals)) in blocking solution and were washed four times with PBS/0.1% Tween20. Incubation with the secondary antibody plus DAPI (1 μg/ml) was also performed in blocking solution for 1 h and the cells were again washed four times.

ATM inhibition experiments: Cells were treated as described above but were additionally supplemented with ethinyldesoxyuridine (EdU, 10 μM) for 30 min and briefly washed twice with PBS before fixation, blocking and incubation with primary antibodies (mouse anti-gH2AX (clone JBW301, Merck) or mouse anti-53BP1 (clone BP13, Merck); rabbit anti-geminin (#10802-1-AP, Proteintech)). EdU staining was performed according to the manufacturer’s protocol following the last washing step after the secondary antibody.

For both experiments, the slides were finally mounted with Vectashield mounting medium (Vector Laboratories), and the cells were inspected using an AxioObserver.Z1 fluorescence microscope with ApoTome and Axiovision Software (Zeiss). DSB repair foci per nucleus were assessed manually using stacked images in maximum intensity projection.

### Single Cell Gel Electrophoresis (Comet Assay)

Exponentially growing cells were treated with ATM inhibitor or DMSO for 2 h, harvested by trypsinization, and resuspended in cold medium again supplemented with inhibitor or DMSO before irradiation with 0 or 6 Gy on ice. Cells were then either incubated for the indicated times at 37°C for repair or directly processed by cold centrifugation and resuspension in cold PBS. Approximately 100 µl of cell suspension containing 60,000 cells was then mixed with 300 µl 4% low melt agarose at 40°C and 100 µl was evenly distributed on a microscope slide using wide bore tips and coverage of agarose with large cover slips. After 5 min on ice, cover slips were removed and slides were incubated in cold lysis buffer (2.5 M NaCl, 100 mM EDTA, 10 mM Tris–HCl pH 10.5, 1% N-lauroylsarcosine, 1% DMSO, and 1% (v/v) Triton X-100) for 1 h. Afterwards, slides were briefly rinsed with cold deionized H_2_O (dH_2_O) and washed 3 times for 5 min in cold electrophoresis buffer (1× TBE; 0.89 M Tris Base, 0.89 M boric acid, and 0.02 M EDTA) before electrophoresis for 30 min at 1 V/cm (33V) in a cold room. After electrophoresis, the slides were washed 3 times in cold dH_2_O, air-dried overnight in the dark, and then stored with dessicant at 4 C until staining. For comet staining, slides were rehydrated for 60 min in cold dH_2_O before the addition of 200 µl staining solution (propidium iodide 10 mg/ml in PBS) directly to the slides. After 30 min, slides were rinsed 3 times with PBS and were again air dried before microscopic analysis using a Zeiss Axioplan 2. Pictures were finally analyzed using ImageJ and the OpenComet plugin. A minimum of 30 comets were assessed per condition.

### DSB Reporter Gene Assay

Exponentially growing HNSCC cells containing stably integrated copies of the previously described GFP-based NHEJ or HR reporter plasmids pGC or pEJ (25, 26) were transfected with an I-SceI expression vector for targeted DSB induction using Fugene HD (Promega). Six hours post-transfection, the medium was exchanged and supplemented with ATM inhibitor or solvent (DMSO), followed by another exchange plus supplementation 24 h post-transfection. At 48 h post-transfection, the cells were harvested and assessed for GFP expression by flow cytometry using a FACS Canto with FACS Diva software (Becton Dickinson). The gating of GFP-positive cells was set according to the negative control (Fugene HD + empty vector). Rates of DSB repair (% GFP-positive cells) were normalized to the respective transfection efficiency of the individual experimental samples as determined by parallel transfection with a GFP-expression vector (pEGFP-N1) and are presented as further normalized to the respective solvent controls.

### Assessment of DNA End Resection

The DNA end resection was assessed by flow cytometric quantification of chromosomally bound RPA in relation to the cell cycle phase. Exponentially growing cells were treated with ATM inhibitor and after 30 min irradiated with 6 Gy. At the time points indicated, the cells were harvested by trypsinization and then pre-extracted by gentle resuspension (wide bore tips) of the cell pellet in 500 µl ice cold PBS/0.1% Triton X-100/1mM DTT, followed by gentle shaking in horizontally placed reaction tubes on ice for 10 min. Afterwards, 1 ml cold PBS/1% BSA/1 mM DTT was added, the tubes were inverted several times, and the pre-extracted cells were collected in a precooled centrifuge (5 min, 400 g). Cells were resuspended (wide bore tips) in PBS/4% formaldehyde and fixed for 10 min at room temperature and afterwards blocked with PBS/0.2% Triton X-100/1% BSA for a minimum of 1 h. The cells were subsequently incubated (1 h; room temperature) with a mouse anti-RPA32 antibody (clone ME34, Santa Cruz) in blocking solution. Afterwards, the antibody was first maximally diluted (1.5 ml final volume) and then twice washed with PBS/0.1% Tween20 before incubation (1 h; room temperature) with the second antibody, followed by dilution and two washing steps. DNA counterstaining was performed using DAPI added to the secondary antibody. Flow cytometric analysis was performed using a MACSQuant10 and MACSQuantify (Miltenyi Biotec) and FlowLogic software (Inivai).

### Data Evaluation

Data analysis was performed using Excel (Microsoft) and Prism 6 (GraphPad). All experiments were performed at least three times, except for the Western blot experiments, which were performed twice using independent extracts. The values presented are mean ± SD. A two-tailed Student’s t-test was used to assess statistically significant differences using Prism 6.

## Results

**Figure 1:**
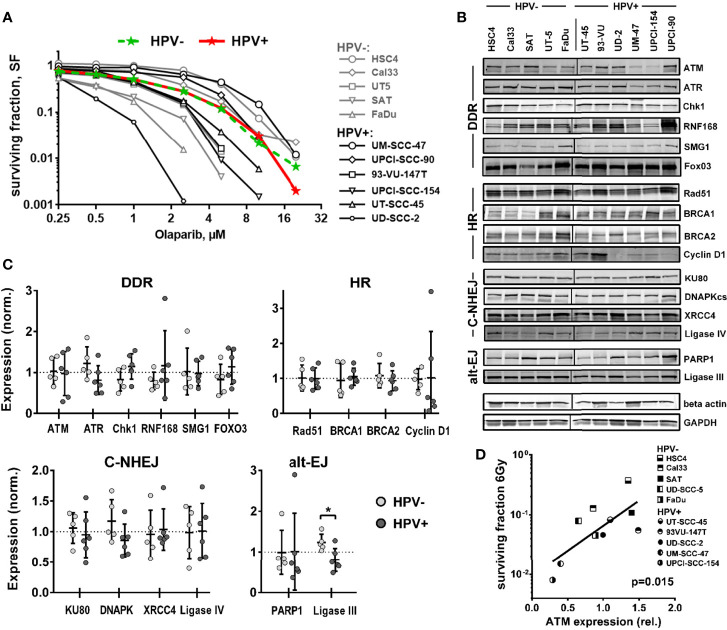
Sensitivity towards PARP inhibition and expression of DNA repair factors. **(A)** Exponentially growing cells were seeded at low, defined numbers and, on the next day, treated with olaparib as indicated. After one week, the medium was exchanged and the cells were incubated without the inhibitor until the formation of colonies. Colored curves represent group means. **(B)** Expression of DNA repair factors in HPV+ and HPV− HNSCC cell lines as determined by Western blot analysis from exponentially growing cells. Note that TRIP12 expression was also tested but no interpretable results were obtained. **(C)** Quantification and comparison of DNA repair factors by HPV status. Individual expression is depicted as normalized to the average expression of the whole cohort (dotted line). An asterisk indicates significance (p <0.05, two-tailed Student’s t-test). **(D)** Association of ATM expression and cell survival in 5 HPV+ and 5 HPV− HNSCC cell lines at 6 Gy as reported previously by us (6).

As outlined above, several different mechanisms have been proposed to cause the enhanced radiation sensitivity of HPV+ HNSCC cells, with defects in HR most frequently suggested. Therefore, we first tested to what extent HPV+ HNSCC cell lines may show hypersensitivity against PARP inhibition, following the well-established concept of synthetic lethality (27, 28). Using a colony formation assay and a panel of 6 HPV+ and 5 HPV− HNSCC cell lines, we observed a high variation of sensitivity in both panels, but on average no difference (**Figure 1A**).

Using the same panel, we next compared the expression levels of central DSB repair factors, including some, whose dysregulated expression was proposed to be responsible for the enhanced radiation sensitivity of HPV+ HNSCC cells (8, 9, 12, 14, 20, 21) by Western blot. No clear differences were observed between the two groups for most proteins tested (**Figures 1B, C** and **Supplementary Figure 1**). One reported finding that could be confirmed was a strongly reduced expression of cyclin D1 in four of six HPV+ strains, albeit due to two outliers with high expression, the group means were similar. The only proteins whose expression was significantly different was the alt-EJ and base excision repair component ligase III, with lower expression being observed in the HPV+ panel. Trends toward reduced expression in HPV+ strains were also observed for ATR and DNAPKcs and toward enhanced expression in HPV+ cells for Chk1, and the latter may to some extent compensate for reduced ATR expression. Though the expression of the central DNA damage response (DDR) kinase Ataxia telangiectasia mutated (ATM) did not differ when looking at the whole panels, a striking observation was the extremely low ATM expression in the HPV-positive cell lines UM-SCC-47 and UPCI-SCC-154, the most radiosensitive strains (6) (**Figure 1D**).

### Influence of ATM on Cell Cycle Arrest, DSB Repair and Survival

**Figure 2:**
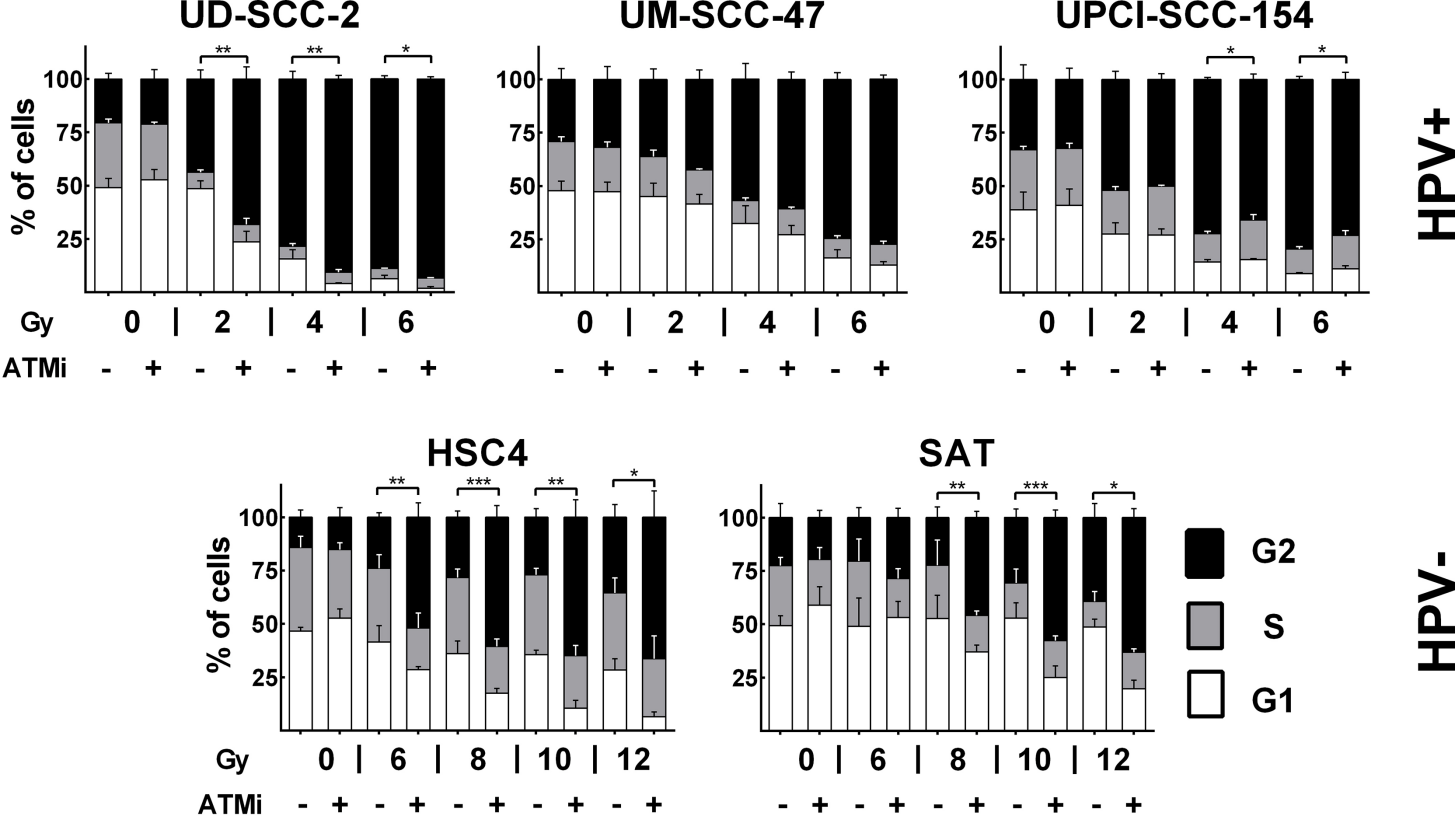
Radiation-induced G2 arrest. Cells were treated for 30 min with the ATM inhibitor before irradiation, as indicated. 24 h after irradiation the cells were fixed and the cell cycle distribution assessed by DAPI staining and flow cytometry. Statistical evaluation was performed for changes in the fraction of G2 phase cells upon ATM inhibition. Asterisks depict significant differences with *, **, and *** indicating p < 0.05, p < 0.01, and p < 0.001 (two-tailed Student’s t-test).

ATM deficient cells, which include cells derived from AT-patients, cells under ATM inhibition, or tumor cells lacking ATM expression, display a severe defect in DSB repair with un- and mis-repaired DSBs leading to high radiosensitivity and a profound and sustained activation of the G2/M cell cycle checkpoint (29–31). Regarding the latter, an especially pronounced radiation-induced G2 arrest was also described for HPV+ HNSCC cells (6, 32). To address whether lack of ATM function may contribute to this phenotype, we tested to what extent an addition of the well established ATM inhibitor KU55933 could further increase the arrest. Strikingly, ATM inhibition had little influence on G2 arrest at 24 h after irradiation in UM-SCC-47 and UPCI-SCC-154, the two HPV+ cell lines with low ATM expression. In the latter, it even resulted in slightly fewer cells in G2 after 4 and 6 Gy. In HPV+ UD-SCC-2 cells, which are also characterized by DSB repair deficiency and extensive G2 arrest but normal ATM levels, an increase was clearly obvious at the lower doses (**Figure 2**). In HPV− HSC4 and SAT cells, where radiation-induced G2-arrest is far less extensive at this late time point, a clear increase was observed upon ATM inhibition at higher radiation doses.

**Figure 3:**
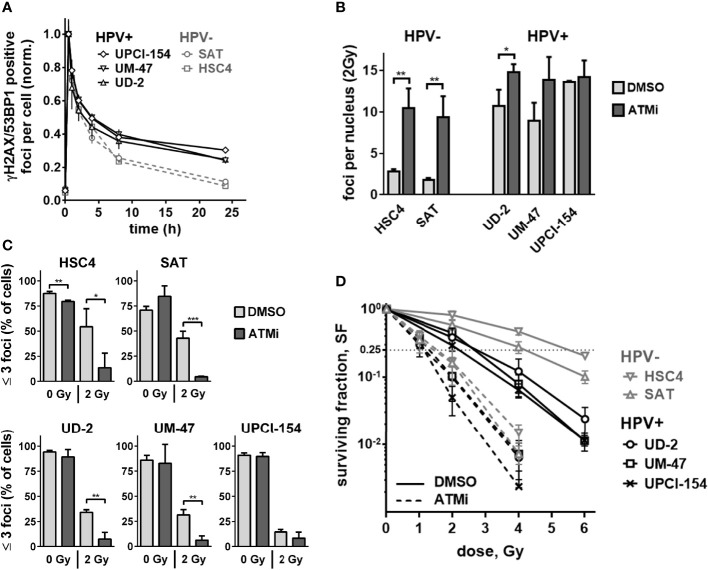
Residual double-strand breaks and radiation sensitivity. **(A)** DSB repair kinetics of HPV+ and HPV− HNSCC cell lines. For each cell line, the values for nuclear foci were normalized to the first time point (30 min) after irradiation with 2 Gy. **(B)** Quantification of nuclear γH2AX foci at 24 h after 2 Gy irradiation with and without ATM inhibition. Counts of the respective non-irradiated controls were subtracted. **(C)** Fraction of cells with three or less nuclear γH2AX foci at 24 h after 0 or 2 Gy. **(D)** Cell survival as determined by colony formation assays with and without ATM inhibitor treatment. In **(B–D)** cells were generally treated with ATM inhibitor or solvent 30 min prior to and for 24 h after irradiation. In **(B, C)**, S phase cells (EdU+, Geminin+) were excluded from quantification. In the case of UM-SCC-47, 53BP1 foci were assessed instead of γH2AX. Statistical evaluation was performed for changes upon ATM inhibition in **(B, C)**, asterisks depict significant differences with *, ** and *** indicating p < 0.05, p < 0.01 and p < 0.001, respectively (two-tailed Student’s t-test).

We and others have shown that ATM deficient cells possess a characteristic DSB repair kinetic after irradiation. A normal, fast initial decline in DSB repair foci numbers is followed by a delayed decline during the slower repair phase, resulting in enhanced numbers of residual DSBs after 24 h (30, 33, 34). Therefore, we next compared the DSB repair kinetics of the three HPV+ and two HPV− strains used above. Again, HPV+ cells demonstrated a phenotype reminiscent of that of ATM-deficient cells when comparing the kinetics of radiation-induced double-positive 53BP1/γH2AX foci to the kinetics of repair-proficient HPV− HSC4 and SAT cells (**Figure 3A**). In these experiments, the repair kinetics of UD-SCC-2 cells, an HPV+, DSB repair defective strain with normal ATM expression level, also demonstrated the ATM-deficiency resembling phenotype. As HPV+ HNSCC cells have also been reported to demonstrate delayed, less effective repair of radiation-induced DSBs in neutral comet assays (9, 12, 19), we compared the repair in HPV+ and HPV− cells in this setting with and without ATM inhibition. We observed a generally rapid repair and no clear impact of HPV-status or ATM-inhibition (**Supplementary Figure 2**). While in part differing from the previous studies, the results are well in line with the initially fast DSB repair foci kinetics shown in **Figure 3A** and the reported repair kinetics of ATM-deficient cells. Since ATM is especially important for the repair of a comparably small subfraction of breaks, assay sensitivity is crucial and likely limited in the direct gel electrophoretic assessment of DSBs (30, 35, 36). We further analyzed the levels of residual DSB foci at 24 h after 2 Gy irradiation with and without ATM inhibition. Here, we quantified γH2AX foci in G1 and G2 phase cells, where γH2AX most reliably detects DSBs and is not also induced through aberrant replication processes. For this purpose, the cells were co-stained with the S phase marker EdU and the S/G2 phase marker geminin (**Supplementary Figure 3A**). For UM-SCC-47 we detected 53BP1 foci because in this strain γH2AX foci are tiny and hard to distinguish from background staining when assessed alone. In line with the theory of an intrinsically compromised ATM-orchestrated DDR, we observed only a modest (on average 1.33 fold) increase of residual γH2AX foci in the HPV+ cell lines as opposed to an expected pronounced increase (on average 4.42 fold) in HPV− HSC4 and SAT cells (**Figure 3B**, **Supplementary Figure 3B**). With regard to the cell cycle, ATM inhibition effectively increased the amount of residual DSBs in G1 and G2 in HPV− cells, whereas the effect in G2 was rather negligible in the HPV+ strains, which already featured high levels of residual DSBs in G2 after IR alone (**Supplementary Figure 3C**). As cells with few or no residual DSBs have the best chances to evade mitotic cell death after irradiation, we also quantified the fraction of cells with ≤3 foci. At 24 h after 2 Gy ATM inhibition profoundly reduced this fraction in all cell lines (**Figure 3C**). In UPCI-SCC-154, which had not demonstrated any increase in overall residual foci numbers (**Figure 3B** and **Supplementary Figure 3B, C**), the decrease was the smallest and failed to reach significance, but still amounted to 43% as compared to the respective DMSO control. On average, ATM inhibition reduced the fraction of cells with ≤3 foci from 48.6 to 9% in the HPV− cells and 26.7 to 7.3% in the HPV+ ones.

As the number of unrepaired DSBs is a major determinant of cellular radiosensitivity (37), the reduced impact of ATM inhibition on DSB repair foci in the HPV+ tumor cells should result in less effective radiosensitization in clonogenic assays compared to the HPV− strains. In fact, ATM inhibition still induced meaningful radiosensitization in all cell lines. The extent, however, was smaller in HPV+ strains with a mean dose enhancement ratio of 2.11 vs. 3.24 at a surviving fraction of 25%. Overall, cell survival between the two groups was more similar after ATM inhibition and was significantly associated with the fraction of cells with low damage levels (**Figure 3D** and **Supplementary Figure 3D**). In the non-irradiated samples, ATM inhibition reduced the average numbers of colonies in the HPV− cell lines by 41.2% and in HPV+ cells by only 17.6%, further suggesting reduced effectiveness of ATM-inhibition in HPV+ HNSCC cells (**Supplementary Figure 4**).

### Influence of ATM Inhibition on DSB Repair Pathways

**Figure 4:**
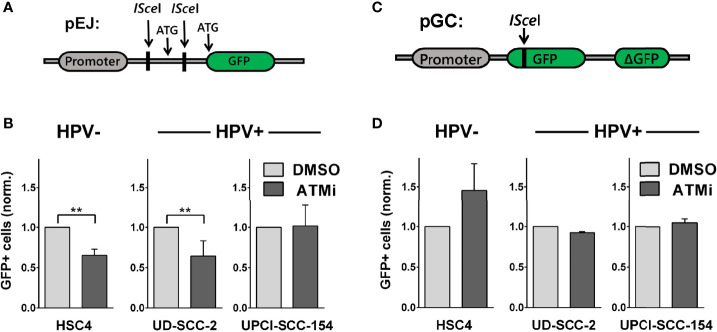
Effect of ATM inhibition on DSB repair pathways. Reporter strains with stable integration of the respective repair constructs were transfected with an I-SceI expression vector. At 6 h and again at 24 h after transfection, the medium was exchanged and the inhibitor added. Forty-eight hours after transfection, the fraction of GFP-positive cells was assessed by flow cytometry. **(A, C)** Schemes of the NHEJ reporter construct pEJ and the HR reporter construct pGC. **(B)** Effect of ATM inhibition on NHEJ efficacy and **(D)** on HR efficacy. Results were normalized to the respective solvent controls of the individual experiments. Statistical evaluation was performed for changes upon ATM inhibition. Asterisks depict significant differences, with ** indicating p < 0.01 (two-tailed Student’s t-test).

We further tested the impact of ATM on the main DSB pathways, NHEJ and HR, using established I-SceI-based plasmid reconstruction assays (25, 26). As a master regulator of DSB detection and processing, ATM interacts with critical components of both pathways. Regarding NHEJ, it, among other functions, mediates the recruitment of 53BP1-RIF1 and the shieldin complex to DSBs and directly phosphorylates 53BP1 (38–40). Assessing NHEJ capacity in subclones stably carrying the pEJ reporter construct (**Figure 4A**), we indeed observed reduced repair efficacy in HSC4 and UD-SCC-2 upon ATM inhibition, in line with excess DSB repair foci in G1 phase, where NHEJ accounts for the vast majority of DSB repair (**Figure 4B** and **Supplementary Figure 3C**). UPCI-SCC-154 was not responsive in the pEJ reporter gene assays and was the only strain not to demonstrate an increase in G1 upon ATM inhibition.

ATM also activates a number of critical HR factors, such as CTIP, Mre11, and Exo1 (38, 41), but data on the requirement of ATM for DSB repair by HR is controversial, with studies suggesting prominent roles (29, 42–44) or independence from ATM (45–47), possibly depending on the cellular context and activity of other PI3K-related kinases. We assessed HR capacity in clones of HSC4, UD-SCC-2, and UPCI-SCC-154 stably carrying the pGC construct (**Figure 4C**). In the HPV+ cells, we did not observe any effect of ATM inhibition on HR efficacy. While this non-responsiveness would be in line with an ATM defect, we also did not observe a reduction but even an increase in HR capacity in HPV− HSC4 cells (**Figure 4D**). Therefore, the data rather suggest that HR at I-SceI-induced, frank DSBs do not prominently depend on ATM in HNSCC in general, rather than providing direct clues about the function of ATM in HPV+ HNSCC. Regarding the radiosensitization under ATM-inhibition (**Figure 3D****),** the pEJ measurements support an impairment of NHEJ inhibition as a contributing factor in HPV− and some HPV+ strains.

### Assessment of ATM-Function

**Figure 5:**
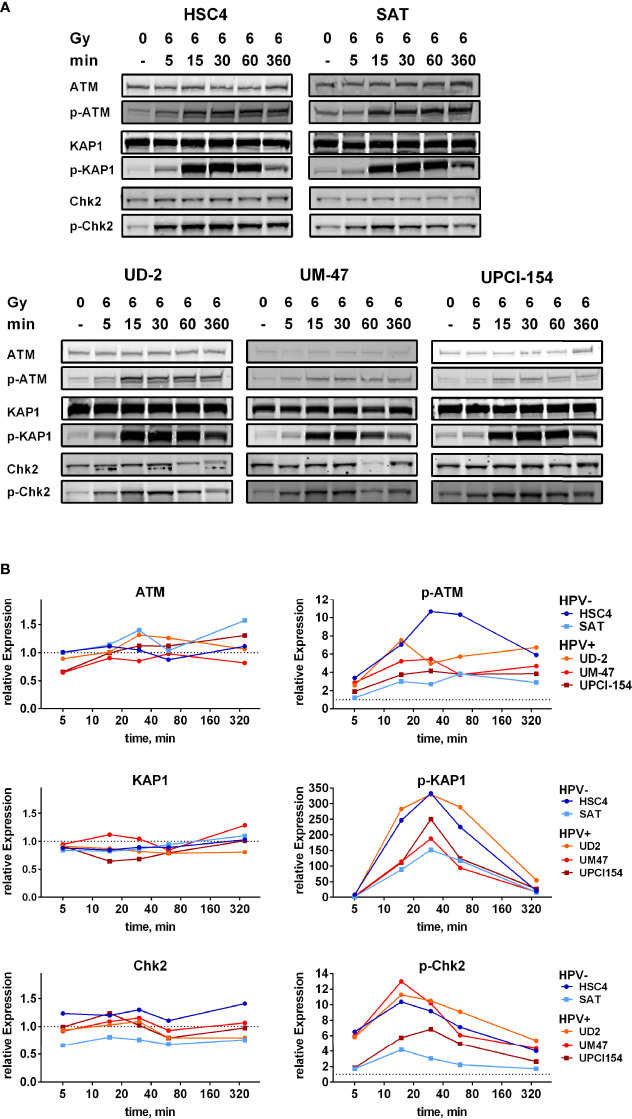
Phosphorylation kinetics of ATM target proteins. Exponentially growing cells were irradiated with 6 Gy and harvested in sample buffer at the indicated time points. **(A)** Western blot signals. **(B)** Quantification. Data points represent the mean of two independent experiments. Left: whole protein level. Right: phosphorylation. Dotted lines indicate the normalized signal intensity of the non-irradiated samples.

To directly test whether ATM function is compromised in radiosensitive HPV+ HNSCC cells, we assessed ATM-mediated signal transduction after irradiation in three ATM target proteins: Chk2 (T68), KAP1 (S824), and ATM itself (autophosphorylation at S1981). Using ATM-inhibition in HSC4 and UPCI-SCC-154, we first confirmed that ATM is indeed the main kinase for phosphorylating these sites (**Supplementary Figure 5**). Since the compromised DSB repair and profound radiosensitization upon ATM inhibition (**Figure 3**) strongly indicate that in HSC4 and SAT the ATM-orchestrated DDR is fully functional, these two cell lines were considered positive controls for sufficient ATM target phosphorylation after irradiation. Intriguingly, on this basis, we did not observe any clear defect in ATM function in the HPV+ strains. While whole protein levels remained stable after radiation, phosphorylation of ATM target sites was clearly induced in all cell lines. The phosphorylation kinetics of all three target sites were overall similar in HPV+ and HPV− strains, and we also did not detect a delay in the ATM-mediated signal transduction as an increase in phosphorylation was mostly evident within 5 min after irradiation (**Figure 5A**). For quantification, the phosphorylation signals were first normalized to their matching whole protein signals and then to the respective untreated controls. We observed generally profound induction of ATM target phosphorylation in HSC4 and a somewhat less effective induction in SAT, which was to some extent due to normalization to higher background levels. The radiation-induced phosphorylation of ATM targets in HPV+ cells was generally at least as effective as that in HPV− SAT cells (**Figure 5B**).

**Figure 6:**
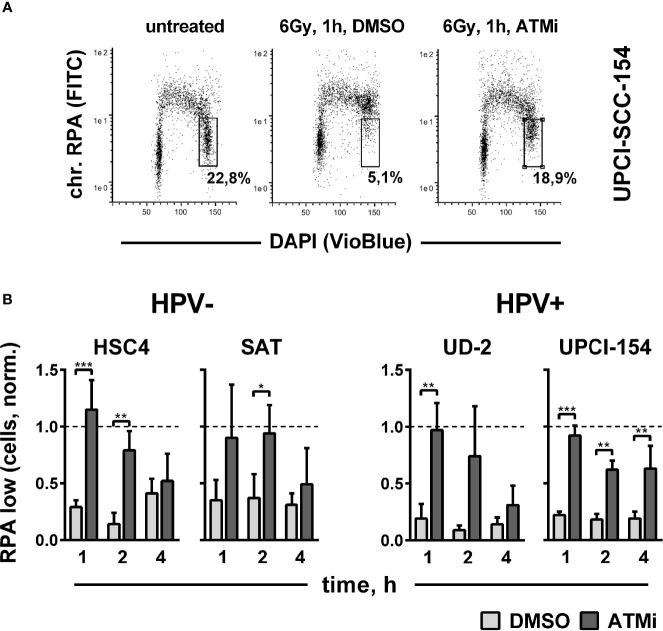
Impact of ATM on radiation-induced DNA end resection. Cells were treated with or without an ATM inhibitor for 30 min before irradiation with 6 Gy. At the indicated time points, cells were pre-extracted before fixation and flow cytometric assessment of chromatin-bound RPA. **(A)** Gating strategy for G2-phase cells with normal (low) amounts of chromatin-bound RPA. **(B)** Quantification of the fraction of G2 phase cells with normal levels of chromatin-bound RPA at the indicated time points after radiation. To reduce variation through differences in the starting fractions of G2-phase cells, values in all individual experiments were normalized to the respective untreated controls harvested at the time of DMSO/ATM inhibitor treatment, which is represented by the dotted line (Y = 1). Asterisks depict significant differences, with *, **, and *** indicating p < 0.05, p < 0.01, and p < 0.001, respectively (two-tailed Student’s t-test).

Though not essential for HR in our reporter gene assay (**Figure 4B**), ATM has been clearly shown to promote DSB end resection through the phosphorylation of critical nucleases (41, 48). Therefore, we finally tested end resection as another endpoint of ATM function mechanistically located between target phosphorylation and residual foci levels. DSB end resection takes place mainly in the S and G2 cell cycle phases and generates stretches of vulnerable single-stranded DNA. Immediate coverage by replication protein A (RPA) is crucial for the protection of these stretches and a necessity for effective resection (49). Later on, RPA is replaced by other factors, such as Rad51, as DSB repair proceeds. We assessed DSB end resection after irradiation by flow cytometric quantification of chromatin-bound RPA after a pre-extraction step to release the unbound fraction. A clear enhancement of chromatin-bound RPA levels was apparent in G2 phase cells at 1 h after irradiation in all tested strains, approaching the high levels of replicating S phase cells (**Figure 6A**). ATM inhibition reduced end resection to a similar extent in the HPV+ and HPV- strains (**Figure 6B**), which, in line with the Western blot results, suggests that ATM is generally functional in HPV+ HNSCC cells.

Since these results indicate the functionality of the ATM itself, it may be possible that factors downstream of ATM are affected in the HPV+ HNSCC cells. The transcription factor p53 is a prominent downstream target of ATM but is known to be phosphorylated and subsequently stabilized/activated upon irradiation also through other kinases such as ATR or DNA-PKcs. p53 is effectively degraded as a function of the HPV16 E6 oncoprotein and it has been suggested that the activation of small, residual amounts of wtp53 contributes to the radiosensitivity of HPV+ HNSCC cells (32). Along this line, a further reduction of p53 activity in HPV+ HNSCC cells through ATM-inhibition could in principle contribute to radioresistance and reduce the otherwise sensitizing effect of ATM-inhibition. However, p53 was hardly detectable in the HPV+ HNSCC cells also 2 and 5 h after irradiation, and there was no clear effect of ATM inhibition on the expression of the p53 downstream effector p21 in both HPV+ and HPV− HSC4 cells harboring mutant p53. Therefore, a meaningful impact of the ATM downstream effector p53 that would explain the reduced responsiveness toward ATM inhibition in HPV+ HNSCC cells appears unlikely (**Supplementary Figure 6**).

## Discussion

Various mechanisms have been proposed to be responsible for the enhanced cellular radiation sensitivity of HPV+ HNSCC cells. Conflicting with the repeatedly reported general HR defect, we observed a high variation but no enhanced sensitivity towards PARP inhibition as compared to HPV− HNSCC cells (**Figure 1A**). Similar results have been reported for another HNSCC panel, including two novel HPV+ strains (50). Furthermore, we could not recapitulate the findings of generally altered protein expression reported for a number of key repair factors, with the exception of cyclin D1 and a non-significant trend for DNA-PKcs (**Figures 1B, C** and **Supplementary Figure 1**). We did, however, observe a significantly reduced expression of ligase III, a similar trend for ATR, and strikingly low expression levels of the central DDR kinase, ATM, in the two most radiosensitive HPV-positive cell lines. The latter observation motivated a search for features of ATM deficiency in 3 radiosensitive and DSB repair deficient HPV+ strains. Our results in this regard are quite controversial. DSB repair foci kinetics and a reduced effectiveness of ATM inhibition regarding (i) radiation-induced G2 arrest, (ii) residual radiation-induced DSB repair foci, especially in G2 phase cells, and (iii) radiosensitization in colony forming assays together speak in favor of a partial impairment in the ATM-mediated DDR (**Figures 2**, **3** and **Supplementary Figure 3**). However, the overall fast and effective phosphorylation of ATM target proteins upon irradiation and the observed reduction of radiation-induced DSB end resection upon ATM inhibition clearly indicate that ATM is generally functional (**Figures 5**, **6**). Confirmative results for this study were obtained in patient-derived *ex vivo* OPSCC slice cultures, where 9 of 11 HPV+ samples tested did not respond to ATM-inhibition on the level of residual DSB repair foci, whereas all 4 HPV− samples tested did (7). While the exact mechanisms are yet to be demonstrated, these results strongly suggest that the DDR anomalies described here represent a specific phenotype and not a cell culture artefact.

Based on these observations, we suggest that in HPV-positive HNSCC cells, factors downstream of ATM may be responsible for the observed lack of effectiveness in the DDR. However, our data do not support a meaningful impact of the downstream factor p53 in this regard, and the identity of potentially impaired downstream factors is yet to be unravelled. It is easily imaginable that a common misregulation in the DDR exists, for example, if directly caused by the functions of the viral oncoproteins E6 or E7. As an example, E7 mediates the degradation of the RB tumor suppressor, which has been directly implicated in DSB repair through cNHEJ (51) and regulation of DSB end resection (52, 53). Regarding ATM, it was recently reported that cells from retinoblastoma patients with germline RB mutations demonstrate radiation sensitivity, presumably through an impact on ATM (54). However, to what extent these mechanisms may be involved in the DSB repair deficiency of HPV+ HNSCC is currently unknown. Furthermore, it is just as imaginable that the formation of HPV-driven HNSCC is favored by a variety of different disturbances in the DDR and DSB repair machinery, with different factors and mechanisms affected in individual tumors. Such heterogeneity would clearly constitute a major challenge for the clarification of the underlying mechanisms and may in part explain the so far partly diverse findings in different studies. Alternative to downstream defects, since ATM phosphorylates numerous targets and exerts multiple functions, our assessment of ATM activity may have missed subtle insufficiencies despite the inclusion of three target sites and time kinetics in the Western analyses and DSB end resection as a further, comparably direct, endpoint. Interestingly, a recent publication reports on two radioresistant subclones of the radiosensitive HPV+ strain UPCI-SCC-154, one of which demonstrates enhanced ATM/P-ATM levels. This indicates that different mechanisms, including a normalization of the ATM protein level, can counteract the high radiosensitivity (55). Liu et al., have described non-responsiveness of HPV+ HNSCC cells towards the TGF-β pathway, which results in a switch from HR to alt-EJ and reduces ATM activity (14). While some of our results are in line with the latter finding, the proposed mechanism of reduced levels of the ATM activating protein FoxO3 caused by enhanced miR-182 expression could not be substantiated in our Western blot comparison (**Figures 1B, C** and **Supplementary Figure 1**). Furthermore, we previously observed no reduction in EJ-mediated repair in plasmid reconstruction assays upon PARP-inhibition in HPV+ HNSCC cells, although PARP inhibition should reduce alt-EJ effectiveness (25). Vitti et al. have performed inhibition of ATM in 2 HPV+ vs. 2 more radioresistant HPV− HNSCC cell lines and, similar to our study, observed effective radiosensitization but to a somewhat larger extent in the HPV− strains. However, this phenotype was not specific to ATM inhibition but was similarly observed for ATR and DNA-PKcs inhibition. On the one hand, the observed radiosensitization points to generally functional repair pathways also in HPV+ cells, but on the other hand a higher effectiveness was observed in HPV− ones and the radiation sensitivity of HPV+ cells is not as strong as one would expect for cells with a complete lack of function of these central DSB repair components. The differences were generally smaller using proton irradiation, which generates more complex DNA damage harder to repair also for HPV- cells (56). Finally, from our point of view, a severe defect in cNHEJ is difficult to reconcile with the effective DSB repair early after irradiation in DSB repair foci kinetics and comet assay in our analysis (**Figure 3A** and **Supplementary Figure 2**), although it should be mentioned that others have observed differences in repair effectiveness in comet assays using similar radiation doses (9, 12, 19).

Our results of an impaired DDR in HPV-positive HNSCC cells further conflict with long-standing knowledge in the virology field. High-risk HPV-types activate ATM and a considerable number of further DDR and DNA repair factors, which is a requirement for effective viral DNA replication during the normal viral life cycle. Amongst other mechanisms, a direct interaction of ATM and the HPV oncoprotein E7 has been described, but its contribution to ATM activation remains unclear (57–59). The discrepancy between an impaired DDR in HPV+ HNSCC cells after irradiation and the activation of the DDR by high-risk HPV during their normal life cycle may suggest that the activation is not so much a direct functional feature of the HPV oncoproteins but to a larger extent a consequence of the rapid and error-prone productive viral DNA replication process (60), which is usually lost in HPV+ malignancies.

Our data strongly suggest that a partial deficiency in the ATM-orchestrated DDR contributes to the DSB repair defect and enhanced radiation sensitivity of HPV-positive HNSCC cells. The exact molecular mechanisms, however, are yet to be discovered. The reduced effectiveness of ATM inhibition concerning radiosensitization of HPV+ cells argues against a clinical exploration of this approach since ATM inhibition also induces severe radiosensitization in non-tumor cells (30, 38), which in sum likely results in an unfavorable therapeutic ratio.

## Data Availability Statement

The original contributions presented in the study are included in the article/**Supplementary Material**. Further inquiries can be directed to the corresponding author.

## Author Contributions

SK, HZ, LK, FG, SC, RR, FM, and TR conducted experiments. SK and TR supervised experiments. SK, HZ, NS, WYM, MK, and TR analyzed the data. SK, KR, CP, CB, and TR contributed conception and design of the study. SK, KR, and TR wrote the manuscript. All authors listed have made a substantial, direct, and intellectual contribution to the work and approved it for publication.

## Funding

This work was supported by the Brigitte und Dr. Konstanze Wegener-Stiftung (SK, TR), the Erich und Gertrud Roggenbuck-Stiftung (SK, TR) and the German Federal Ministry of Education and Research (BMBF, grant 02NUK032; WYM, MK, KR, TR).

## Conflict of Interest

The authors declare that the research was conducted in the absence of any commercial or financial relationships that could be construed as a potential conflict of interest.

## Publisher’s Note

All claims expressed in this article are solely those of the authors and do not necessarily represent those of their affiliated organizations, or those of the publisher, the editors and the reviewers. Any product that may be evaluated in this article, or claim that may be made by its manufacturer, is not guaranteed or endorsed by the publisher.
