# Supplementary material for: A Lack of Effectiveness in the ATM-Orchestrated DNA Damage Response Contributes to the DNA Repair Defect of HPV-Positive Head and Neck Cancer Cells
Source: Front Oncol. 2022 May 31;12:765968. doi: 10.3389/fonc.2022.765968 (PMC9204973; doi:10.3389/fonc.2022.765968)
Supplement: Supplementary file 1 [file DataSheet_1.pdf]

Supplementary Material

Antibody	Clone or Order Number	Manufactorer
rabbit monoclonal anti-ATM	D2E2 / #2873	Cell Signaling
goat polyclonal anti-ATR	#sc-1887 (N-19)	Santa Cruz
mouse monoclonal anti-Chk1	2G105 / #2360	Cell Signaling
rabbit polyclonal anti-RNF168	#ABE367	Merck
rabbit polyclonal anti-SMG1	#4993 (Q25)	Cell Signaling
rabbit monoclonal anti-FoxO3a	D19A7 / # 2829	Cell Signaling
rabbit monoclonal anti-Rad51	#PC130	Merck
mouse monoclonal anti-BRCA1	D-9 / #sc-6954	Santa Cruz
rabbit monoclonal anti-BRCA2	#A 303-434A	Bethyl
mouse monoclonal anti-Cyclin D1	DCS-6 / #AH10148	Abgent
rabbit monoclonal anti-Ku80	#2753	Cell Signaling
rabbit polyclonal anti-DNA-PK	#PC127/#D54962	Merck
rabbit polyclonal anti-XRCC4	#AHP387	Bio-Rad
mouse monoclonal anti-Ligase IV	D-89/ #sc271299	Santa Cruz
rabbit monoclonal anti-PARP1	46 D11 / #9532	Cell Signaling
mouse monoclonal anti-Ligase III	clone 7 / #611876	BD
mouse monoclonal anti-β-Actin	AC-15 / #A1978	Merck
mouse monoclonal anti-GAPDH	6C5 / #32233	Santa Cruz
rabbit monoclonal anti-phospho-ATM (Ser1981)	#EP1890Y	Epitomics
rabbit polyclonal anti-KAP1	#10484	Abcam
rabbit monoclonal anti-phospho-KAP1 (Ser824)	#EPR5248	Abcam
mouse monoclonal anti-Chk2	Clone 19/#611570	BD
rabbit polyclonal anti-phospho-Chk2 (Thr68)	#2661	Cell Signaling
mouse monoclonal anti-p53	Clone DO7	Novocastra
rabbit polyclonal anti-p21	#2947	Cell Signaling
goat anti-mouse IgG IRDye 680RD	#926-68070	LI-COR
goat anti-rabbit IgG IRDye 680	#926-68071	LI-COR
goat anti-rabbit IgG IRDye 800	#926-32211	LI-COR

Supplementary Table 1: Antibodies used in Western blot experiments.

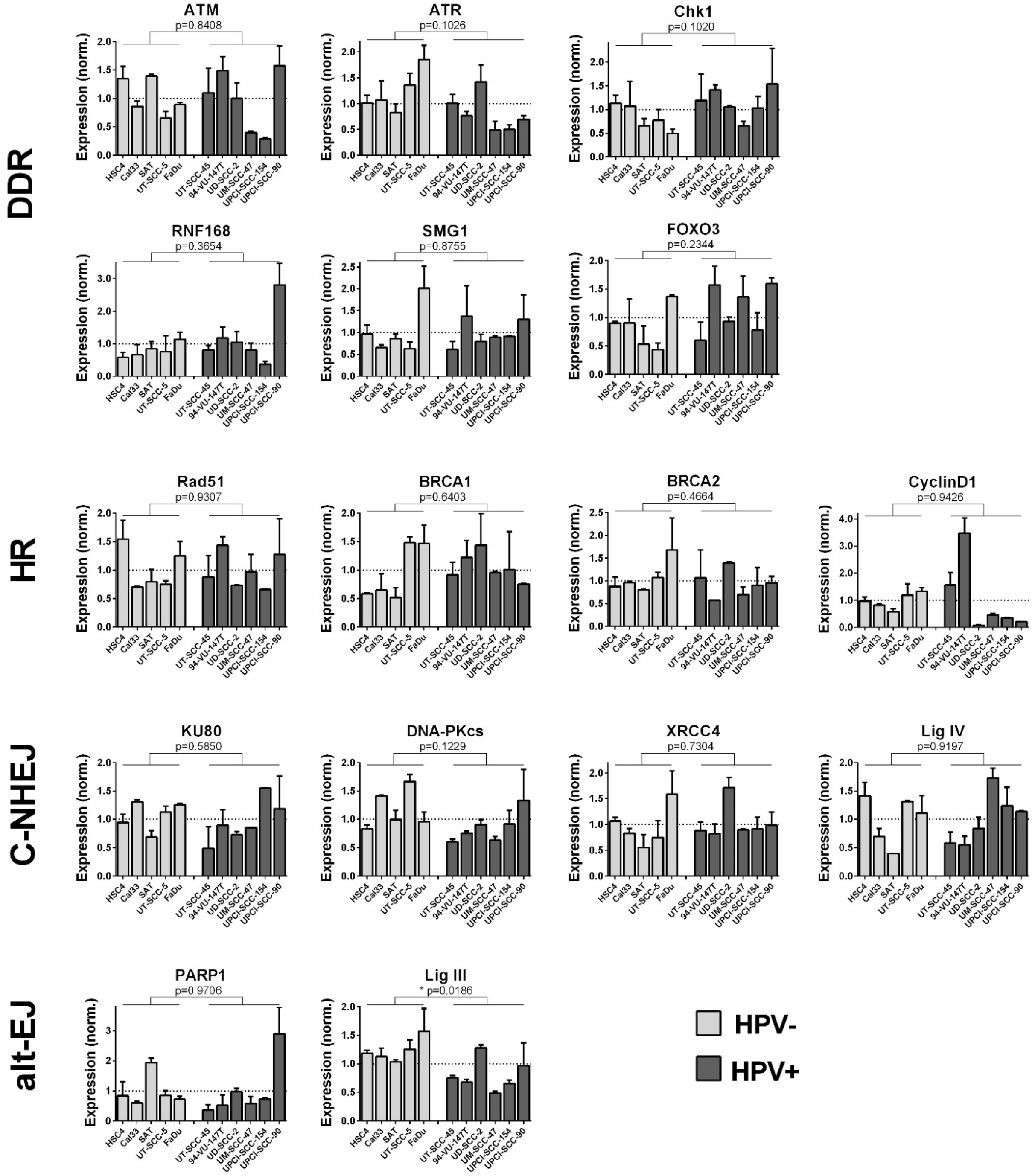

Supplementary Figure 1. Expression of DSB repair factors. Expression of DNA repair factors in individual HPV+ and HPV- HNSCC cell lines as determined by Western blot analysis from exponentially growing cells. Graphs are based on the same data as subfigures 1B,C.

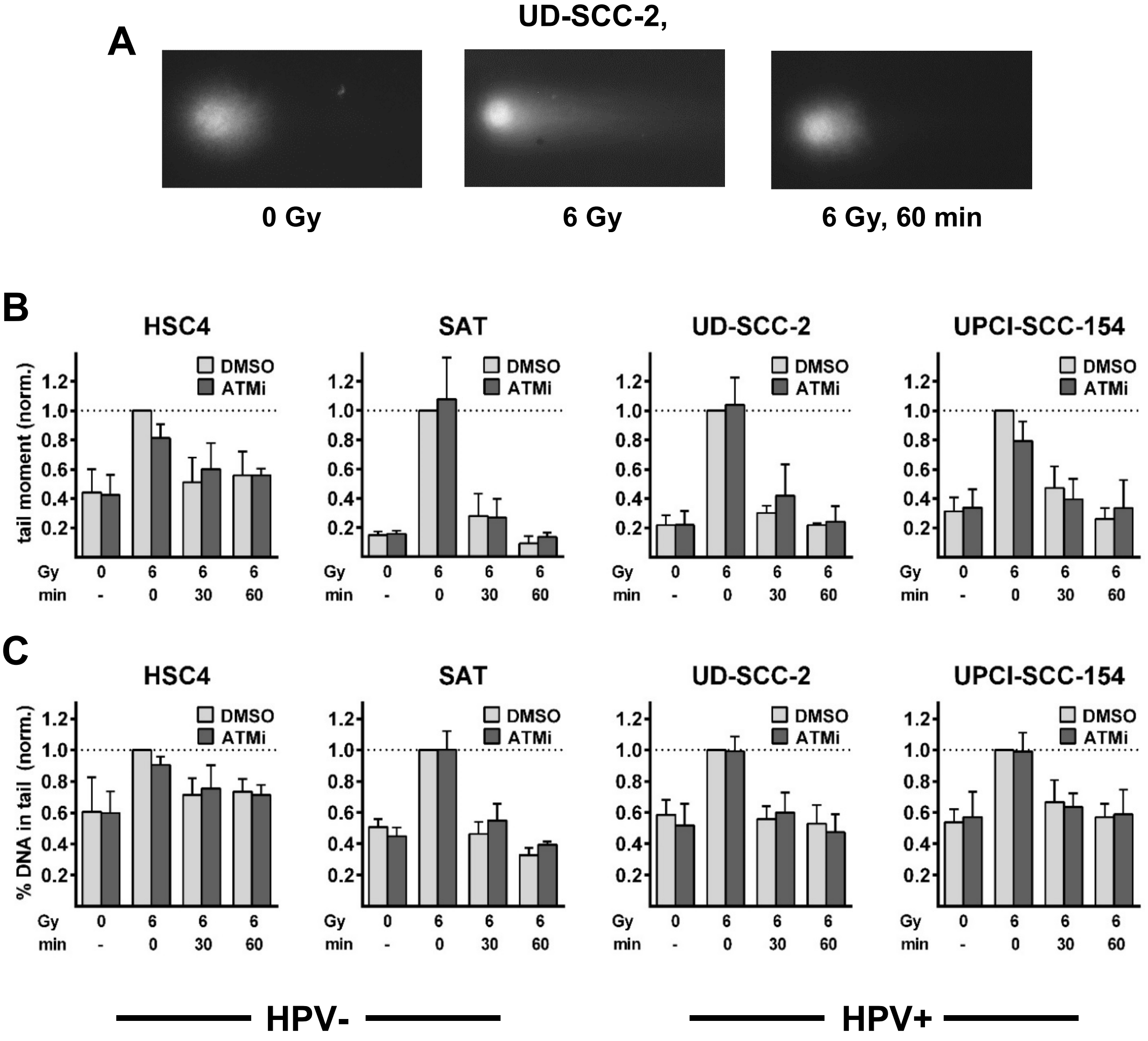

Supplementary Figure 2. Comparison of DSB repair in single cell gel electrophoresis assays. DSB repair as assessed in neutral comet assays. Cells in suspension were treated with DMSO or ATM inhibitor, irradiated with the indicated doses on ice and afterwards granted the indicated repair time at 37°C. A) Examples. B) Quantification. Results from individual experiments were normalized to the tail moment of irradiated, DMSO treated cells immediately processed after irradiation. Reduced induction of tail moment in HSC4 cells is mainly due to a higher background level in non-irradiated cells.

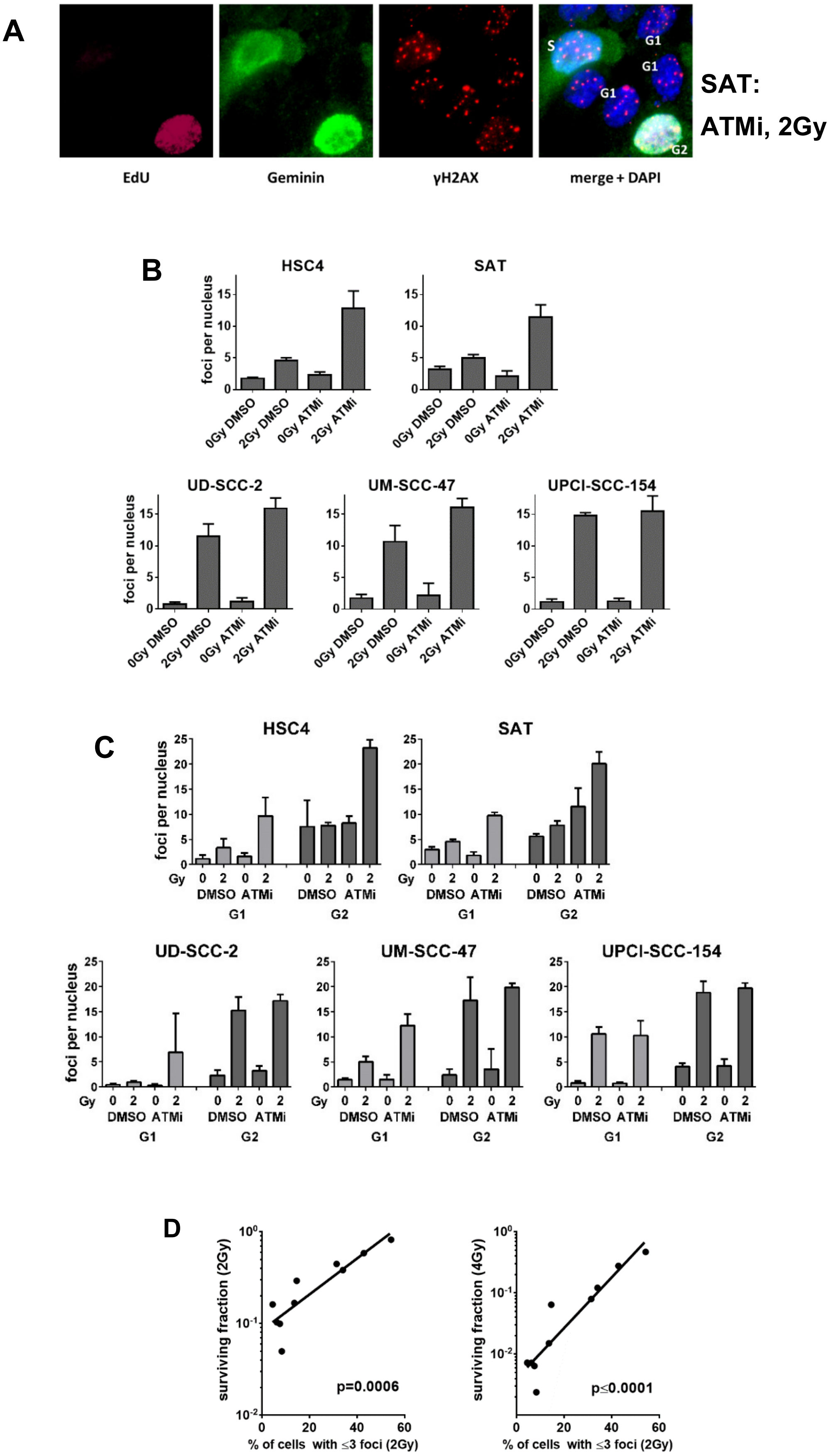
A
EdU
Geminin
γH2AX
merge + DAPI
S
G1
G1
G1
G2
SAT:
ATMi, 2Gy
B
HSC4
SAT
UD-SCC-2
UM-SCC-47
UPCI-SCC-154
foci per nucleus
0Gy DMSO
2Gy DMSO
0Gy ATMi
2Gy ATMi
C
HSC4
SAT
UD-SCC-2
UM-SCC-47
UPCI-SCC-154
foci per nucleus
Gy
DMSO ATMi
G1
G2
D
surviving fraction (2Gy)
surviving fraction (4Gy)
p=0.0006
p≤0.0001
% of cells with ≤3 foci (2Gy)

Supplementary Figure 3. Residual double-strand breaks. Exponentially growing cells were treated with ATM inhibitor KU55933 (10 μM) and irradiated as indicated. 24h after irradiation, cells were pulsed with EdU (30 min), fixed and stained for γH2AX, geminin and EdU. Only cells in G1 (EdU-, geminin-) or G2 (EdU-, geminin+) were scored. In the case of UM-SCC-47 53BP1 foci were assessed instead of γH2AX. Graphs represent the same experiments as Figure 3B,C. **(A)** Staining example (SAT, 2Gy, ATMi). **(B)** Quantification of nuclear γH2AX foci in irradiated and non-irradiated cells. **(C)** Quantification of nuclear γH2AX foci in irradiated and non-irradiated cells with respect to the cell cycle phase. **(D)** Association of the surviving fraction and the fraction of cells with a maximum of 3 residual DSB repair foci after 2 Gy irradiation. Each dot represents the mean values of an individual cell line treated with DMSO or ATMi.

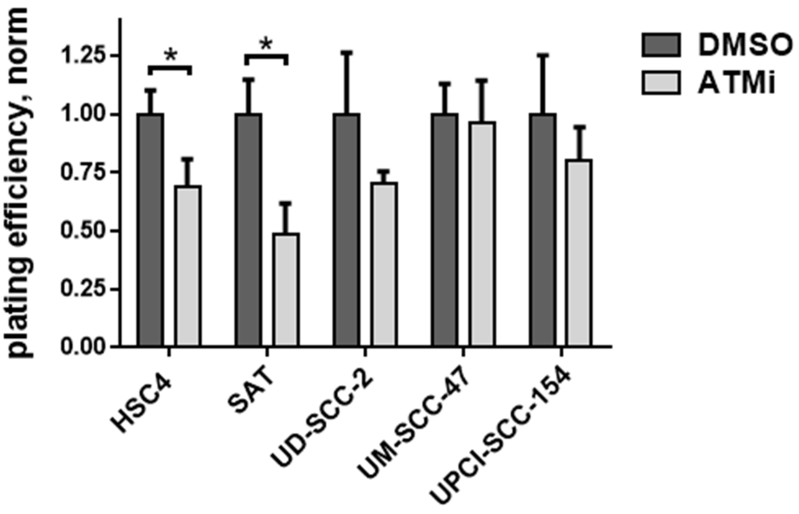

Supplementary Figure 4. Plating efficiencies of colony formation assays. Exponentially growing cells were treated with or without KU55933 (10 µM). After 24.5 h the cells were seeded in defined, low cell numbers without inhibitor for colony formation. Graph represent the surviving fractions of the 0 Gy samples from the experiments shown in Figure 3D. Single values were normalized to the mean DMSO value of the respective experiment, bars represent the mean of these normalized values + SD. Statistical evaluation was performed for changes upon ATM inhibition, asterisks depict significant differences with * indicating $p<0.05$ (two-tailed Student's t-test).

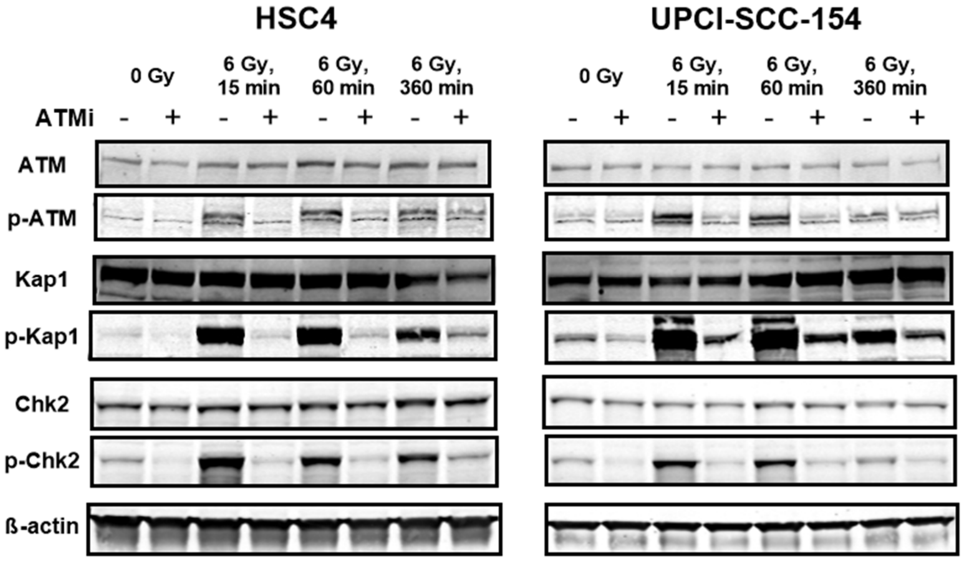

Supplementary Figure 5. ATM specific phosphorylation of target proteins. Exponentially growing cells were treated with and without KU55933 (10µM), irradiated with 6 Gy and harvested in sample buffer at the indicated time points.

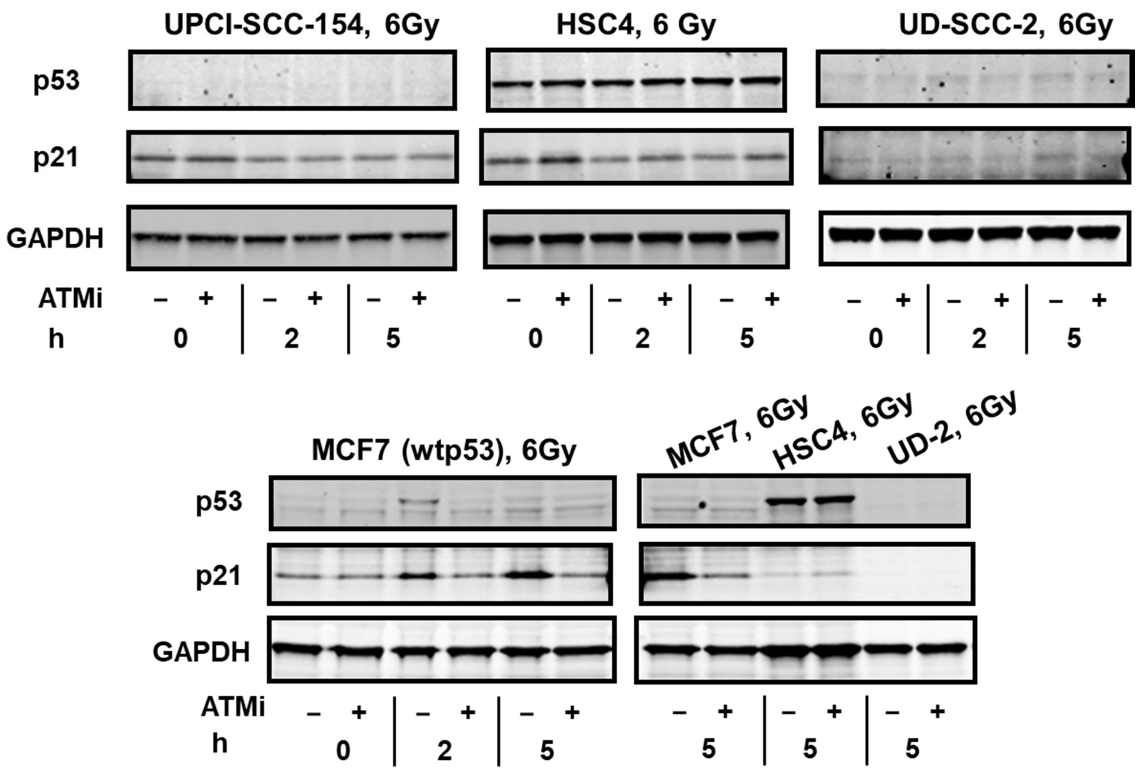

Supplementary Figure 6. p53 and p21 expression after irradiation. Exponentially growing cells were treated with and without ATM inhibitor, irradiated with 0 or 6 Gy and harvested in sample buffer after 2 and 5 h as indicated.UPCI-SCC-154 and HSC4 are directly comparable as bands are derived from the same blot. HSC4 cells harbor non-functional mutant p53, which is generally expressed at high levels. MCF7 breast cancer cells were utilized as a non-HPV-derived wtp53 cancer cell line for comparison and as a positive control for p53-induced p21 induction. Note the induction of p53 expression in MCF7 at 2 h past irradiation without ATM inhibition, which has already decreased at 5 h and the longer lasting p21 response. 5 h samples of MCF7, HSC4 and UD-SCC-2 were further directly compared on the same blot.
